# Antihyperlipidemic, Antihyperglycemic, and Liver Function Protection of *Olea europaea* var. Meski Stone and Seed Extracts: LC-ESI-HRMS-Based Composition Analysis

**DOI:** 10.1155/2021/6659415

**Published:** 2021-03-17

**Authors:** Amina Ben Saad, Mohamed Tiss, Henda Keskes, Anisa Chaari, Maria Eleni Sakavitsi, Khaled Hamden, Maria Halabalaki, Noureddine Allouche

**Affiliations:** ^1^Laboratory of Organic Chemistry, Natural Substances Team (LR17-ES08), Faculty of Sciences of Sfax, University of Sfax, PB “1171”, PC “3000”, Sfax, Tunisia; ^2^Laboratory of Bioresources: Integrative Biology and Exploiting, Higher Institute of Biotechnology of Monastir, University of Monastir, Tunisia; ^3^Division of Pharmacognosy and Natural Products Chemistry, Department of Pharmacy, National and Kapodistrian University of Athens, Panepistimiopolis, Zografou, 15771 Athens, Greece

## Abstract

Methanol and methanol/water extracts of olive stones and seeds from *Olea europaea* var. meski were analyzed by reversed-phase high-performance liquid chromatography (HPLC) with diode array detection and mass spectrometry (LC-MS/MS). A total of 28 metabolites were identified; among them are hydroxycinnamic acid derivatives, phenolic alcohols, flavonoids and flavonoid glucosides, secoiridoids, and terpenes. All the extracts were screened for the inhibitory effect of key enzymes related to diabetes and obesity, such as *α*-amylase and lipase. An *in vitro* study revealed that *Olea* meski stone ethanol (MSE) and methanol (MSM) extracts and *Olea* meski seed ethanol (MSE1) and methanol (MSM1) extracts exert an inhibitory action against lipase and *α*-amylase. The most potent activity was observed in the StM extract with IC_50_ equal to 0.19 mg/ml against DPPH oxidation, 1.04 mg/ml against *α*-amylase, and 2.13 mg/ml against lipase. In HFFD rats, the findings indicated that the increase of body weight, LDL, TC, and glucose levels and then the decrease in HDL-C were significantly suppressed in the MSM-treated group than those in HFFD rats. Moreover, the MSM extract exhibited a prominent selective inhibitory effect against intestinal lipase and *α*-amylase activities. The MSM extract was also able to protect the liver-kidney functions efficiently, which was evidenced by biochemicals and histological studies.

## 1. Introduction

The value of *Olea europaea* L. has a long history in traditional medicine, especially in Mediterranean countries. Different parts of the olive tree as well as its products mainly olive oil and olive drupes have been used for centuries for their nutritional properties but also for their health protective effects. Recent research works confirm this valuable profile. Olive drupes have been known to reduce blood sugar, cholesterol, and uric acid. It has also been used to treat diabetes, hypertension, inflammation, diarrhea, respiratory and urinary tract infections, stomach and intestinal diseases, asthma, hemorrhoids, and rheumatism and as a laxative, mouth cleanser, and vasodilator [1]. These biological effects are mainly attributed to certain phenolic compounds found in the *Olea* genus such as phenylethanols, secoiridoids, flavonoids, and lignans [2–4].

Specifically, olives and their byproducts are recognized as a valuable source of natural phenolic antioxidants [2]. For instance, secoiridoid derivatives exhibit a diverse range of biological properties [5–8]. Oleuropein, the major secoiridoid of olive fruit and leaves, has been assessed for its antioxidant potential, anti-inflammatory, antimicrobial [9], and antiviral activities [10]. Recent reports demonstrated an antiamyloidogenic effect of oleuropein suggesting a possible protective role against Alzheimer's disease [11]. Regarding composition, several studies have been carried out mainly focusing on the phenolic composition in olive oil, the most well-known and studied product of olive tree. Much less is the information on the phenolics of other *Olea* organs such as olive fruits, stones, stems, and roots [12, 13]. According to the literature, little attention has been taken for *Olea europaea* var. meski stone and seed extracts leading to a limited knowledge about their phytochemical content.

In the current work, the chemical composition of different extracts (ethyl acetate, methanol, and methanol/water) of olive stones and seeds from *Olea europaea* var. meski using ultrahigh-performance liquid chromatography coupled with the high-resolution mass spectroscopy (UPLC-MS) technique was studied for the first time. In parallel, the effect of these extracts on key enzymes related to obesity, hyperlipidemia, and hyperglycemia and liver-kidney functions was undertaken.

## 2. Materials and Methods

### 2.1. Collection of Plant Material

**Figure 1:**
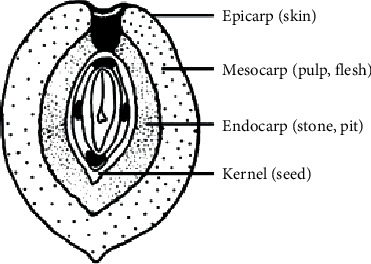
Olive fruit organs.

The seeds (kernel) and stones (wood shell and endocarp) (Figure 1) of olive fruits from *Olea europaea* var. meski were subjected to extraction by maceration in solvents of increasing polarity: ethyl acetate, methanol, and methanol/water (80/20). This extraction procedure led to the production of six extracts (Table 1).

### 2.2. Animal Study

Male Wistar rats, with body weights of 180 to 200 g and bred in the Central Animal House and obtained from the Central Pharmacy, Tunisia, were used in this study. The animals were fed on a pellet diet (Socco, Sfax, Tunisia) and water ad libitum. The animals were maintained in a controlled environment under standard conditions of temperature and humidity with an alternating light-and-dark cycle. The handling of the animals was approved by the Tunisian Ethical Committee for the care and use of laboratory animals.

### 2.3. Experimental Protocol

A total of 32 rats were randomly subdivided into four experimental groups (4 × 8) and subjected to the following treatment in a period of 90 days: the C0 group—control rats fed on a standard diet (control diet (CD) group) at the start of the experiment as a reference; the C90 group—control rats fed on a standard diet (control diet (CD) group) for 90 days; the HFFD group—rats fed on high-fat high-fructose diet (HFFD) composed of 59.95% normal diet, 20% sheep fats, 20% fructose, and 0.5% cholic acid for 90 days; and HFFD + MSM group—rats fed on HFFD and received additional 100 mg/kg of body weight of MSM extract in a volume of 1 ml water daily for 90 days.

After the end of the period of 90 days, the serum was obtained from the trunk and collected after decapitation. Plasma was immediately separated by centrifugation at 4°C and 1,500 × g for 15 minutes. The samples were stored at −80°C until further use. The intestine of each rat was excised and the lumen was flushed out several times with 0.9% NaCl. The scraped off mucosal tissue was pooled, homogenized, and centrifuged at 4,000 × g, and the supernatant was frozen until use in further enzymatic assays.

### 2.4. Analytical Methods

The activities of intestinal lipase and *α*-amylase activities were determined using commercial kits from Biomagreb (Tunis, Tunisia). The activities of aspartate and alanine transaminases (AST and ALT) and lactate dehydrogenase (LDH) as well as the levels of total cholesterol, triglyceride, and HDL cholesterol in the serum were measured using commercial kits from Biomagreb (Tunis, Tunisia) and BIOLABO (Lyon, France).

### 2.5. UHPLC-ESI-HRMS and HRMS/MS Analyses

A Waters ultra-performance liquid chromatography (UPLC) system hyphenated to a hybrid LTQ Orbitrap Discovery mass spectrometer (Thermo Scientific, Brehmen, Germany) was employed for the analysis. The mass spectrometer was equipped with an electrospray ionization (ESI) source and operated in a negative mode. The UPLC system is composed by a Waters pump and a Waters autosampler. Stock solutions of 300 *μ*g/ml (ACN/water 1 : 1, *v*/*v*) of all the extracts were prepared and 10 *μ*l was injected on a Fortis C18 (Fortis Technologies) column (2.1 x 100 mm, 1.7 *μ*m). The mobile phase used was aqueous acetic acid 0.1% (*v*/*v*) (solvent A) and methanol (solvent B). The initial conditions were 95% of solvent A and 5% of solvent B, adjusting the gradient to 100% B in 20 min. This solvent composition was maintained for 1 min (100% B) followed by a return to the initial conditions and a re-equilibration step (3 min) prior the next run. The flow rate was set to 400 *μ*l/min. The mass tolerance was set to 5 ppm. MS Data were acquired in the ESI(−)mode, in the full-scan *m*/*z* range of 115–1000 with a resolution of 30,000. MS2 spectra were recorded using data-dependent acquisition with a CID value of 35% and a mass resolution of 7,500. Capillary temp was set at 350°C with a respective source voltage set on 2.7 kV. Tube lens and the capillary voltage were tuned at −100 and −30 V in the negative mode. Finally, nitrogen was used as sheath gas (40 arbitrary units) and auxiliary gas (10 arbitrary units). Xcalibur 2.0.7 software was used for the pre- and postacquisition of the results.

## 3. Results

### 3.1. Phytochemical Analysis

*Olea europaea* var. meski was cultivated in Tunisia and its detailed phytochemical content is not yet reported. The current study provides the LC-HRMS and HRMS/MS profiling of methanol and methanol/water extracts from olive stones and seeds. Interestingly, the phytochemical analysis of the aforementioned plant material revealed differences as a matter of quality and relative quantity of the identified secondary metabolites in the two plant organs. Specifically, chromatographic and spectrometric features such as retention time and HRMS/MS data allowed the detection and identification of 28 secondary metabolites as well as the performance of a comparative study for the presence of these constituents in the different parts of woody endocarp of olive fruits. The suggested elemental composition (EC) was based on the pseudomolecular ions, the HRMS/MS fragments, and the respective indicative RDBeq values which assisted drastically in the identification process. Moreover, the electrospray ionization (ESI) method, in the negative mode, was chosen due to its efficiency for the detection of secondary metabolites in olive fruits.

**Figure 2:**
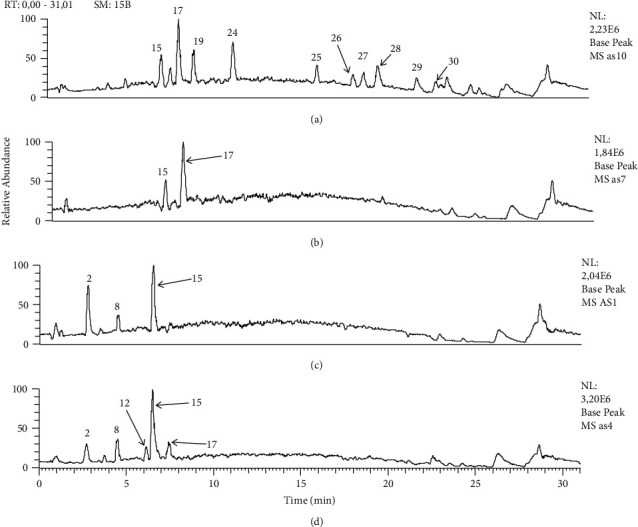
Comparative base peak chromatograms of all extracts from major identified metabolites, *Olea europaea* var. meski organs. (a) Stones (methanol); (b) stones (methanol/H_2_O); (c) seeds (methanol); (d) seeds (methanol/H2O). 2: hydroxytyrosol; 8: oleoside 11-methyl ester; 12: verbascoside; 15: nuzhenide; 17: oleuropein; 19: ligstroside; 24: 9,12,13-trihydroxy octadeca-7-enoic acid; 25: oleuropein aglycon; 26: maslinic acid; 27: hydroxyoctadecadienoic acid; 28: oleanolic acid; 29: eicosanoic acid; 30: dihydroxyheneicosanoic acid.

Noticeably, olive stones are the richest in secondary metabolites among the studied olive organs particularly for the methanol extract. 31 metabolites were identified; among them are phenolic alcohols, hydroxycinnamic acid derivatives, secoiridoids, flavonoids, terpenes, and fatty acids. Verbascoside (*m*/*z* 623.19629) was the main hydroxycinnamic acid derivative identified in all extracts with the main fragment at *m*/*z* 461.1638. Two simple phenyl alcohols hydroxytyrosol (*m/z* 153.05582) and its glycosylated derivative (*m*/*z* 315.10764) were detected only in *Olea* meski seed extracts. Hydroxytyrosol was more abundant in the methanol extract. Likewise, the glycosylated derivative of tyrosol, salidroside (*m*/*z* 299.11270), was detected in the two investigated olive organs. The main secoiridoid derivatives, oleuropein (*m*/*z* 539.17572) and ligstroside (*m*/*z* 523.18121), were found in olive stones and seeds, as long as they are hydrolyzed on glycosidic bond derivatives oleuropein aglycon (*m*/*z* 377.12378) and ligstroside aglycon (*m*/*z* 361.12854), respectively. Oleuropein was more abundant in the two olive stone extracts. The monoterpene iridoid, nuzhenide (*m*/*z* 685.23309), was found in all extracts in a significant amount and was more abundant in olive seeds. Similarly, neonuzhenide (*m*/*z* 701.22791) was also detected in all extracts but in a less abundance. Regarding flavonoids, luteolin (*m*/*z* 285.03976) and its glycoside derivatives luteolin*7-O-*rutinoside (*m*/*z* 593.15009) and luteolin hexoside (*m*/*z* 447.09238) were detected only in olive seeds with a weak abundance. The typical olive triterpenes, maslinic (*m*/*z* 471.34674) and oleanolic (*m*/*z* 455.35178) acids, were identified only in the olive stones and they were more abundant in the methanol extract. On the other hand, four fatty acids (9,12,13-trihydroxy octadeca-7-enoic acid, hydroxyoctadecadienoic acid, eicosanoic acid, and dihydroxyheneicosanoic acid) were identified only in the methanol extract of the olive stones (Table 1 and Figure 2).

### 3.2. Effect of Methanol, Hexane, and Water Olive Byproduct Extracts on *α*-Amylase and Lipase Activities In Vitro

Table 2 showed that olive byproduct extracts (methanol, hexane, and aqueous) showed dose-dependent lipase and *α*-amylase inhibition. The methanol olive stone extract (MSE) exhibited the lowest IC_50_ of 1.04 mg/ml against *α*-amylase and IC_50_ of 2.13 mg/ml against lipase. The standard positive controls acarbose and atorvastatin showed an IC_50_ of 0.39 and 1.1 mg/ml, respectively (Table 3).

### 3.3. Intestinal Lipase Activity and Plasma Lipid Concentration

**Figure 3:**
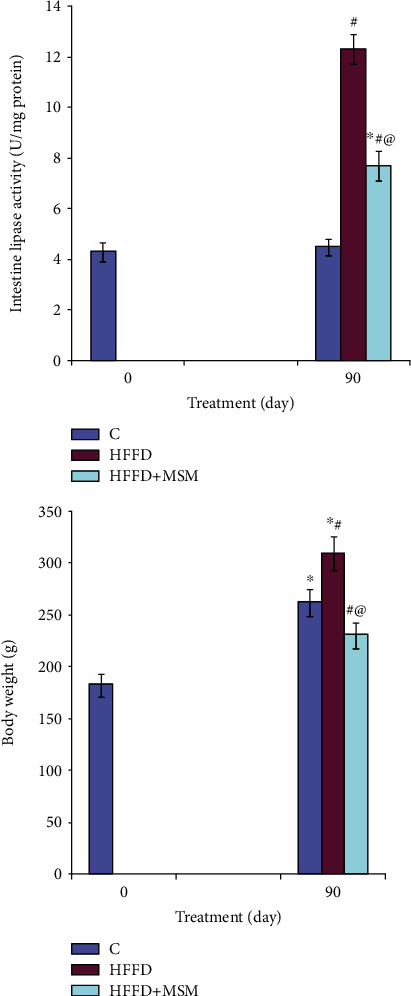
Intestinal lipase activity and body weight in HFFD rats treated with MSM. ^∗^*P* < 0.05 significant differences compared to controls (0 day). ^#^*P* < 0.05 significant differences compared to controls (90 days). ^@^*P* < 0.05 significant differences compared to HFFD rats.

Figure 1 indicates that the administration of MSM extract to HFFD rats reverted the activity of lipase in the intestine back by 37%. This supplement was also observed to bring about a considerable decrease of 17 and 48% in the TC and LDL-C concentrations, respectively, in the plasma. Moreover, while diabetes was noted to induce a decrease in the HDL-Ch concentration by 51%, the MSM supplement was observed to prevent this decrease (Figure 3 and Table 2).

### 3.4. Intestinal *α*-Amylase Activity and Plasma Glucose Levels

**Figure 4:**
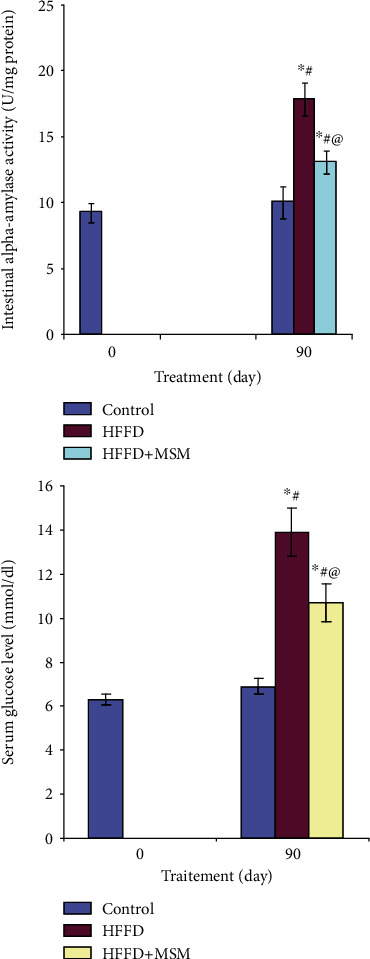
Intestinal *α*-amylase activity and blood glucose level in HFFD rats treated with MSM. ^∗^*P* < 0.05 significant differences compared to controls (0 day). ^#^*P* < 0.05 significant differences compared to controls (90 days). ^@^*P* < 0.05 significant differences compared to HFFD rats.

Figure 2 shows that the supplementation of MSM extract to HFFD rats was found to significantly decrease the activities of *α*-amylase by 26%. In addition, the administration of MSM to HFFD rats reduced the glucose concentration in plasma by 27% as compared to HFFD-untreated rats (Figure 4).

### 3.5. Hepatic Function, HFFD Diet, and MSM Extract

**Figure 5:**
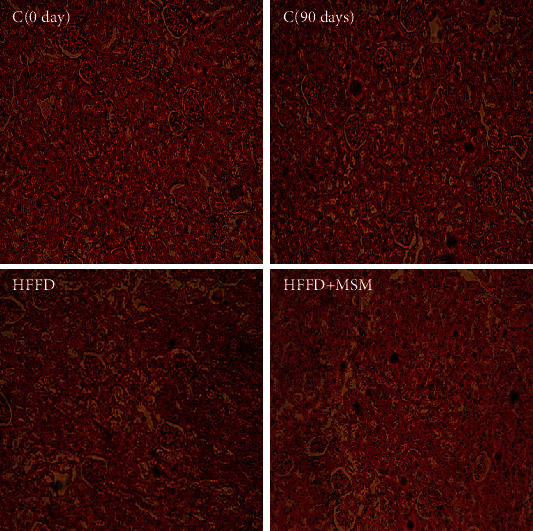
C_0day_ and C_90days_: normal rat liver; HFFD rat liver: sinusoidal congestion and fatty degeneration in the form of fat lake; HFFD + MSM: diabetic treated with MSM—a positive effect was observed (H&E 100).

In HFFD rats treated with MSM extract, a clear protective effect was observed in hepatic function and metabolism. In fact, administration of MSM extract inhibited the increase of AST, ALT, and LDH in plasma of HFFD. This positive effect of this extract was confirmed by histological finding. As shown in Figure 4, fatty cysts appeared in hepatic tissues of HFFD rats. However, MSM administration to HFFD rats reduced the appearance of fat cells in the liver (Table 2 and Figure 5).

## 4. Discussion

In this study, Table 1 summarizes the results of the profiling study including some spectroscopic characteristics and major fragments of the detected compounds. All the ECs and the fragmentation patterns agree with the previous reports [1, 12]. The chemical composition of olive fruits and olive oil has been extensively studied while other *Olea* organs such as stems, roots, and drupe stones have received little attention leading to a limited knowledge about their phytochemical content. Indeed, recent studies related to the phytochemical investigation of olive leaves have been referred [14–16], while only few reports are available for olive drupe parts: stones and seeds [13]. According to the literature, the main components of olive roots and stems are hydroxytyrosol, tyrosol, oleuropein, and ligstroside [17] as well as verbascoside and flavonoids such as taxifolin, luteolin, and apigenin derivatives (in stem) [18]. However, the chemical composition of stone and olive extracts obtained by methanol and ethyl acetate revealed great similarities in comparing the chemical composition of other olive organs in other studies [17, 18]. Finally, a new insight into the diverse biochemical pathways in the whole tree is gained contributing to the better understanding of the nutritional and medicinal values of olive tree products.

### 4.1. *In Vitro* Study

The present study revealed that the extracts obtained from *Olea* meski seeds and stones using methanol or methanol/H_2_O mixture possess remarkable inhibitory activity on lipase and *α*-amylase. The most potent inhibitor effect was observed in the methanol extract from *Olea* meski stone (MSM) extract. This is would be explained by the presence of several metabolites well known by their antidiabetic and antihyperlipidemic activities such as oleuropein, oleuropein aglycon, and oleanolic acid [19, 20].

In HFFD rats, this study showed that the administration of MSM extract to HFFD rats inhibits key enzymes related to lipid digestion and absorption as lipase in the intestine and consequently to a decrease in TC and LDL-C and to an increase in HDL-C as compared to normal rats. In fact, phytochemical analysis showed that MSM extract contains bioactive metabolites such as hydroxytyrosol, oleuropein, oleuropein aglycon, and oleanolic acid which are known by their antihyperlipidemic effects and also by their inhibitory activity against lipase. A previous study has demonstrated that oleuropein decreased the body weight gain in vivo [21]. In this context, Park and coworkers [22] have established that oleuropein, at low concentration, attenuated hepatic steatosis induced by high-fat diet in mice [22]. More recent reports have shown that oleuropein supplementation at high concentration (0.59%) significantly decreased the body weight, decreased leptin concentration, and modulated gene expression related to obesity in mice [23].

Furthermore, our findings are in agreement with the report of Hamden and coworkers who demonstrated that flavonoids and polyphenols are able to suppress enzymes related to lipid absorption and consequently to show antiobesity and antihyperlipidemic effects [2]. In addition, it was shown in a previous investigation that administration of hydroxytyrosol-rich extract (3 mg/kg) for 17 weeks in Wistar rats fed a cholesterol-rich diet attenuated the increase in serum triglycerides and total and LDL cholesterol [24].

In addition, this study showed that administration of MSM to HFFD rats inhibited intestinal *α*-amylase activity and reduced the blood glucose level as compared to untreated HFFD rats. Our results are in agreement with the report of Tiss et al. [25, 26] who induced diabetes in male Wistar rats via intraperitoneal injections of alloxan monohydrate (150 mg/kg), and animals with hyperglycemia (blood glucose levels of 2 g/l after 2 weeks) were retained for experimentation. The treatment groups were given daily hydroxytyrosol by gastric gavage route which resulted in significantly decreased blood glucose levels. The hepatic toxicity indicators TBARS, bilirubin, and fatty cysts were reduced in animals receiving HT treatment. Hepatic glycogen, circulating high-density lipoprotein (HDL), and antioxidant enzymes (SOD, CAT, and GPX) in the liver and kidney were increased by HT. On the other hand, we have noticed that the increase of glucose and lipid levels caused the formation of free radicals of oxygen (ROS). The oxidative atmosphere in cells caused the damage of cells and tissues in the liver. Moreover, the oxidative atmosphere that attacks the hepatic and kidney function appeared by an increase in the levels of AST, ALT, and LDH, an indication of liver dysfunction. However, both the hypoglycemic and hypolipidemic actions of MSM prevented glucose and lipid toxicities as ROS formation. In fact, this study showed that the MSM extract exhibited a strong promising potential as a protective therapeutic agent against liver and kidney toxicities in diabetic rats. It proved remarkably efficient in the decrease of liver dysfunction indices in HFFD rats, namely, the AST, ALT, and LDH activities confirmed by histological analysis.

## 5. Conclusion

In conclusion, this study indicated that *Olea* meski stone played an effective role in the amelioration of type 2 diabetes by inhibition of *α*-amylase activity and the decrease of body weight and normalization of the lipid profile. In addition, *Olea* meski stone protects liver-kidney-heart functions and tissues.

## Figures and Tables

**Table 1:** Secondary metabolites detected in the different *Olea europaea* meski stone and seed extracts.

No.	Rt (min)	Proposed EC	[M − H]^−^*m*/*z*	*Δ* in ppm	RDBeq	*m*/*z* MS^2^ (Relative intensity %)	Tentative identification	*Olea* meski stone extracts		*Olea* meski seed extracts	
								MeOH	MeOH/H_2_O	MeOH	MeOH/H_2_O
1	2.54	C_14_H_19_O_8_	315.10764	−2.87	5.5	153.0506 (100), 287.1157, 196.7752	*Hydroxytyrosol hexoside*	−	−	+	+
2	2.77	C_8_H_9_O_3_	153.05582	0.64	4.5	123.0452 (100)	*Hydroxytyrosol*	−	−	++	+
3	3.27	C_14_H_19_O_7_	299.11270	0.57	5.5	Nd	*Salidroside*	+	+	+	+
4	3.45	C_10_H_13_O_5_	213.07648	−1.73	4.5	151.0763 (100), 196.8127, 183.0658	*Hydroxylated products of the decarboxylated form of hydroxy elenolic acid(isomer I)*	+	+	+	+
5	3.55	C_10_H_13_O_5_	213.0765	−1.52	4.5	151.0763 (100), 196.8127, 183.0658	*Hydroxylated products of the decarboxyl elenolic acid (isomer ΙI)*	+	+	+	+
6	3.63	C_11_H_13_O_6_	241.07126	−2.08	5.5	Nd	*Elenolic acid*	+	−	−	−
7	3.78	C_16_H_21_O_11_	389.10782	−2.86	6.5	345.1174 (100), 287.1134, 196.8236	*Oleoside*	−	−	+	+
8	4.50	C_17_H_23_O_11_	403.12350	−2.67	6.5	223.0603 (100), 359.1330, 333.0810, 179.0557.	*Oleoside 11-methyl ester*	+	+	++	++
9	5.82	C_25_H_31_O_14_	555.17032	−0.482	10.5	537.1587 (100), 456.8495, 403.1225, 393.1173, 287.0841	*Hydroxyoleuropein*	+	+	+	+
10	5.90	C_31_H_41_O_18_	701.22791	−1.40	11.5	539.1738 (100), 377.1226, 307.0786, 175.0913	*Neo-nuzhenide*	+	+	+	+
11	5.98	C_21_H_19_O_11_	447.09225	−2.30	12.5	Nd	*Luteolin hexosides*	−	−	+	+
12	6.12	C_29_H_35_O_15_	623.19629	−2.97	12.5	461.1638 (100)	*Verbascoside*	+	+	+	+
13	6.17	C_29_H_35_O_15_	623.19672	−2.29	12.5	461.1639 (100)	*Isoacteoside*	+	+	+	+
14	6.47	C_25_H_35_O_13_	543.20715	−2.13	8.5	525.1948 (100), 513.1946	*Dihydro oleuropein*	−	−	+	+
15	6.51	C_31_H_41_O_17_	685.23309	−1.06	11.5	523.1719 (100), 453.1376, 421.1480, 299.1130	*Nuzhenide*	++	++	+++	+++
16	7.14	C_10_H_11_O_4_	195.06624	−0.22	5.5	Nd	*Hydroxytyrosol acetate*	+	−	−	−
17	7.52	C_25_H_31_O_13_	539.17572	−2.39	10.5	377.1223 (100), 307.0809, 275.0913	*Oleuropein*	+++	+++	+	+
18	7.63	C_25_H_27_O_13_	535.14655	1.55	12.5	Nd	*Comselogoside (p-Coumaroyl-6 oleoside)*	−	−	+	+
19	8.35	C_25_H_27_O_13_	523.18054	−2.97	10.5	361.1273 (100), 291.0858, 259.0962	*Ligstroside*	++	+	+	+
20	8.43	C_19_H_21_O_7_	361.12854	−2.03	9.5	291.0861 (100), 259.0966	*Monoaldehydic form of ligstroside aglycon*	+	+	+	+
21	8.81	C_15_H_9_O_6_	285.03976	−2.44	11.5	Nd	*Luteolin*	−	−	+	+
22	8.87	C_17_H_19_O_6_	319.11795	−2.38	8.5	Nd	*Oleacein*	+	−	+	+
23	8.99	C_25_H_35_O_13_	381.15448	−2.65	7.5	363.1430 (100), 349.1275, 331.1170, 287.121	*Hydroxytyrosol acyclodihydroelenolate*	−	−	+	+
24	10.64	C_18_H_33_O_5_	329.23254	0.289	2.5	229.1438 (100), 311.2214, 293.2111, 211.1334	*9,12,13-trihydroxy octadeca-7-enoic acid*	++	−	−	−
25	15.61	C_19_H_25_O_8_	377.12372	−1.25	9.5	345.0978 (100), 307.0822, 275.0924	*Oleuropein aglycon*	+	−	−	−
26	17.50	C_30_H_47_O_4_	471.34674	−1.47	7.5	423.3267 (100), 405.3052, 393.3250	*Maslinic acid*	++	+	−	−
27	18.11	C_18_H_31_O_3_	295.2378	0.39	3.5	251.2372 (100), 277.2162, 155.1440	*Hydroxyoctadecadienoic acid*	++	−	−	−
28	21.01	C_30_H_47_O_3_	455.35178	−2.83	7.5	196.7925 (100), 287.0947, 407.3293	*Oleanolic acid*	++	+	−	−
29	21.19	C_20_H_37_O_2_	309.1732	−0.41	2.5	287.1234 (100), 196.7988	*Eicosanoic acid*	++	−	−	−
30	22.21	C_21_H_37_O_4_	353.2667	−1.35	4.5	287.0779 (100), 196.8279	*Dihydroxyheneicosanoic acid*	++	−	−	−
31	25.61	C_27_H_29_O_15_	593.15009	−1.86	13.5	Nd	*Luteolin-7-O-rutinoside*	−	−	+	+

Rt: retention time; EC: elemental composition; RDBeq: ring double bond equivalent; [M–H]^−^: *m*/*z* of the pseudomolecular ion; ^+,++,+++^relative abondance; ^−^not detected.

**Table 2:** AST, ALT, and LDH activities and TC, LDL-C, and HDL-C in plasma levels in control, HFFD, and HFFD rats treated with MSM.

	Control (0 day)	Control (90 days)	HFFD	HFFD + MSM
AST	30.7 ± 3.5	36.1 ± 4.6	76.1 ± 9.6^∗#^	47.7 ± 7.44^∗#@^
ALT	35.3 ± 7	41.3 ± 3.7	64.3 ± 5.9^∗#^	52.1 ± 9.1^∗#@^
LDH	19.5 ± 1.9	21 ± 2.1	31 ± 1.9^∗#^	27.9 ± 4.2^∗#@^
TC (g/l)	1.21 ± 0.12	1.34 ± 0.12	1.68 ± 0.12^∗#^	1.39 ± 0.11^∗@^
LDL-C (g/l)	0.56 ± 0.07	0.65 ± 0.08	1.29 ± 0.05^∗#^	0.66 ± 0.09^∗@^
HDL-C (g/l)	0.65 ± 0.08^∗^	0.69 ± 0.06	0.39 ± 0.06^∗#^	0.73 ± 0.05^#@^

^∗^ *P* < 0.05 significant differences compared to controls (0 day). ^#^*P* < 0.05 significant differences compared to controls (90 days). ^@^*P* < 0.05 significant differences compared to HFFD rats.

**Table 3:** Antilipase and anti *α*-amylase inhibition activities (% of inhibition) of methanol, hexane, and water olive byproducts extracts (IC_50_, mg/ml) at different concentrations (*n* = 3).

	IC_50_ (mg/ml)		
Olive byproduct extracts	DPPH	*α*-Amylase	Lipase
Methanol olive stone extract (MSE)	0.19 ± 0.01	1.04 ± 0.10	2.13 ± 0.13
Methanol/water olive stone extract	0.27 ± 0.03	1.94 ± 0.19	3.1 ± 0.11
Methanol olive seed extract	0.25 ± 0.09	1.56 ± 0.17	2.57 ± 0.11
Methanol/water olive seed extract	0.37 ± 0.09	2.37 ± 0.19	4.50 ± 0.19
Standard ascorbic acid	0.19 ± 0.09		
Standard acarbose	0.011 ± 0.02	0.39 ± 0.07	
Standard atorvastatin			1.1 ± 0.10

## Data Availability

Data sharing is not applicable to this article as no datasets were generated or analyzed during the current study.
